# Effect of viewing distance on object responses in macaque areas 45B, F5a and F5p

**DOI:** 10.1038/s41598-022-18482-4

**Published:** 2022-10-03

**Authors:** I. Caprara, P. Janssen

**Affiliations:** 1 Laboratorium Voor Neuro-en Psychofysiologie, Katholieke Universiteit Leuven, Leuven, Belgium; 2 The Leuven Brain Institute, Leuven, Belgium; 3 Department of Neurosurgery, Department of Massachusetts General Hospital, Harvard Medical School, Boston, United States

**Keywords:** Premotor cortex, Sensory processing, Object vision

## Abstract

To perform tasks like grasping, the brain has to process visual object information so that the grip aperture can be adjusted before touching the object. Previous studies have demonstrated that the posterior subsector of the Anterior Intraparietal area is connected to area 45B, and its anterior counterpart to F5a. However, the role of area 45B and F5a in visually-guided grasping is poorly understood. Here, we investigated the role of area 45B, F5a and F5p in object processing during visually-guided grasping in two monkeys. We tested whether the presentation of an object in near peripersonal space activated F5p neurons more than objects with the same retinal size presented beyond reachable distance and conversely, whether neurons in 45B and F5a—which may encode a purely visual object representation—were less affected by viewing distance when equalizing retinal size. Contrary to our expectations, we found that most neurons in area 45B were object- and viewing distance-selective, and preferred mostly Near presentations. Area F5a showed much weaker object selectivity compared to 45B, with a similar preference for objects presented at the Near position. Finally, F5p neurons were less object selective and frequently Far-preferring. In sum, area 45B—but not F5p– prefers objects presented in peripersonal space.

## Introduction

Different brain areas in the dorsal visual stream and its target areas in frontal cortex contribute to object grasping. Although neurons in parietal area V6A also respond during object grasping^1–3^, the most studied parieto-frontal network for controlling the hand comprises the Anterior Intraparietal Area (AIP), the ventral premotor cortex (PMv) and primary motor cortex (M1). AIP neurons are selective for real-world objects^12^, grip type^4^, 3D-^5^ and 2D images of objects and very small fragments^6,7^). Overall, the target areas of AIP in frontal cortex seem to have similar properties. Neurons in the anterior subsector of PMv (F5a), which is connected to 3D-shape selective sites in the anterior subsector of AIP (aAIP—^8^), respond selectively to images of 3D objects^9^ and during object grasping. Visual-dominant neurons (i.e., responding during object fixation but not during grasping in the dark) are present in F5a^9^ but not in F5p^10,11^. A subset of neurons in the posterior part of PMv (F5p) are selective for real-world objects, even during passive fixation^11^. In area F5p, objects are represented mainly in terms of the grip type used to grasp the object^12^. Instead, area 45B, in the anterior bank of the lower ramus of the arcuate sulcus and receiving input from 3D-shape selective sites in the posterior subsector of AIP (pAIP—^8^), responds selectively to 2D images of objects and to very small contour fragments, as in pAIP^13^. Other parts of AIP—not necessarily 3D-shape selective—are also connected to the F5p subsector in PMv^14^.

Few studies have investigated the neural representation of space at the single-cell level in parietal^15,16^ and frontal cortex^17,18^. Lesion studies in monkeys^19^ and humans^20^ have indicated the existence of distinct networks processing near and far space^21^ have shown that visual stimuli in near space activate temporal, parietal, prefrontal and premotor areas, whereas stimuli in far space produced activations in a different network spanning occipital, temporal, parietal, cingulate and orbitofrontal cortex, as suggested by human studies^22,23^. To date, no study has investigated the other two subsectors of area F5 (F5a, F5p) and area 45B concerning space processing.

We investigated the neuronal selectivity for objects and viewing distance in three frontal areas receiving input from AIP. To test whether objects appearing within reachable distance activated these frontal neurons more than when they appeared in extrapersonal space, we presented different objects at two viewing distances while keeping their retinal image size constant. To maximize the activation of a motor plan, we randomly interleaved fixation trials and grasping trials when the object was presented in peripersonal space without informing the animals.

## Materials and methods

### Subjects and surgery

Two adult male rhesus monkeys (D, 7 kg and Y, 12.5 kg) served as subjects for the experiments. All experimental procedures, surgical techniques, and veterinary care were performed in accordance with the NIH Guide for Care and Use of Laboratory Animals and in accordance with the European Directive 2010/63/EU and were approved by the local ethical committee on animal experiments of the KU Leuven.

**Figure 1:**
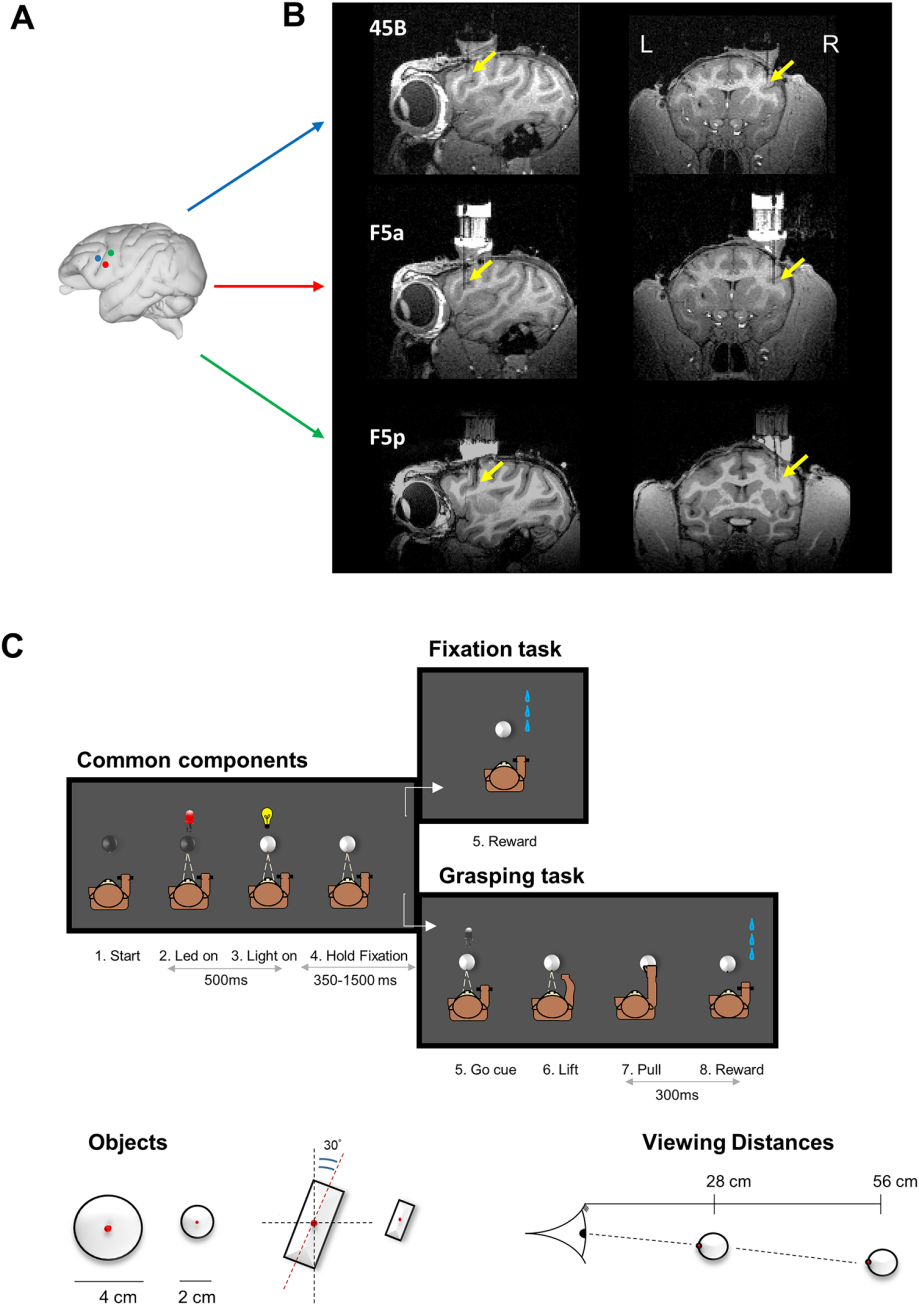
Anatomical location of areas of interest and eye traces. (**A**) Schematic view of a macaque brain (edited from the ‘Scalable Brain Atlas’ http://link.springer.com/content/pdf/10.1007/s12021-014-9258-x). Colored dots represent the areas of interest in the arcuate sulcus, respectively Area 45B—blue, Area F5a—red—and Area F5p—green. (**B**) Anatomical electrode recording position in Monkey D. Yellow arrows indicate the electrode tip location in the three areas. Equivalent recording positions in Monkey Y were reported (data not shown). (**C**). Schematic representation of the behavioral task, stimuli and distances. The first block represents the common events to both Fix and Grasp tasks. The right-upper block corresponds to Fix task, the right-lower represents the Grasp task. Objects: large and small spheres and plates (oriented at 30° from the vertical plane—y-axis; plates size: 5 × 2 cm and 2.5 × 1 cm, respectively). Two viewing distances (Near, Far) from the monkey’s eyes. See details in the text.

An MRI-compatible head fixation post and recording chamber were implanted during propofol anesthesia using dental acrylic and ceramic screws above the right arcuate sulcus in Monkey D and over the left arcuate sulcus in Monkey Y. The recording chamber allowed us to access 45B, F5a and F5p, as shown on MR images with a microelectrode in one of the recording positions for each area (Fig. 1A,B). In a separate test, we measured the binocular eye traces (monkey Y only) when the animal was fixating an LED on the object at the two viewing distances (Supplementary Fig. 1A). The difference in the horizontal eye position between left and right eye was clearly larger at the Near viewing distance compared to the Far viewing distance, indicating that the monkey converged correctly on the object at the two distances. The difference in vergence angle between the two viewing distances was also present in the monocular eye traces obtained during the recordings (Supplementary Fig. 1B).

### Apparatus and Recording procedures

During the experiments, the monkey was seated upright in a chair with the head fixed. Each animal was trained not to move the arm ipsilateral to the recorded hemisphere during the whole duration of the session. In front of the monkey, an industrial robot (Universal Robots, model UR-6-85-5-A) picked up the to-be-grasped object from a placeholder and presented it to the monkey. Four different objects (small plate 2.5 × 1 × 3.2 cm, large plate 5 × 2 × 6.5 cm, small sphere 2 cm diameter, large sphere 4 cm diameter) were pseudo-randomly presented one at a time in front of the monkey. Both plates were oriented at an angle of 30 deg from the vertical plane, therefore allowing the animals to keep the same hand orientation during their grasping. The object could appear either at a Near position (28 cm viewing distance, at chest level ∼ 20 cm reaching distance measured from the center of the hand rest position to the center of the objects—peripersonal space) or at a Far position (56 cm—extrapersonal space—Fig. 1B,C). The average object luminance was: large sphere 3.3 cd/m^2^; small sphere 11.2 cd/m^2^; large plate and small plate 3.4 cd/m^2^. Since both object size and viewing distance were exactly two times larger at the Far position compared to the Near position, the retinal size of a small object at the Near position and the same-shaped large object at the Far position was identical.

The objects required different types of grasping depending on their size, but comparable across monkeys: a pad-to-side grip (for the small sphere and the small plate), and a finger-splayed wrap, corresponding to a whole-hand grip (for the large sphere and the large plate—^24^. Fiber-optic cables detected the resting position of the hand, the start of the reach to grasp movement, and the pulling of the object. The start of the hand movement was detected as soon as the palm of the hand was 0.3 cm above the resting plane, whereas pulling of the object was detected when the object was pulled for 0.5 cm in the horizontal axis.

We recorded single-unit activity with standard tungsten microelectrodes (impedance, 1 MΩ at 1 kHz; FHC) inserted through the dura by means of a 23-gauge stainless-steel guide tube and a hydraulic microdrive (FHC). Neural activity was amplified and filtered between 300 and 5000 Hz. Spike discrimination was performed online using a dual time-window discriminator and displayed with LabView and custom-built software. Spiking activity and photodiode pulses (corresponding to the onset of light in the object, see below) were sampled at 20 kHz on a DSP (C6000 series; Texas Instruments, Dallas, TX). We continuously monitored the position of the left eye with an infrared-based camera system (Eye Link II, SR Research), sampling the pupil position at 250 Hz.

### Experimental Design and Statistical Analysis

The two monkeys were trained to perform two tasks in a dark room, a passive fixation (Fix trials) and a visually guided grasping (VGG, Grasp trials—described in detail in^25^) task (Fig. 1C). In Fix trials, the monkey had to passively fixate a small LED on the object which appeared either at the Near or at the Far distance until he received a juice reward. In Grasp trials, instead, he had to first fixate the LED on the object presented at the Near distance, and then, after a visual go cue (the offset of the LED on the object), to lift the hand from the resting position and pull the object in order to get the reward. Both types of tasks were performed using a robot, which picked one object at the time from a wooden placeholder, and presented it in front of the monkeys at one of the two distances. The fixation period was identical in the two types of trials (350–1500 ms).

To start both the Fix and Grasp trials, the monkey had to place the hand contralateral to the recording chamber in the resting position in complete darkness. During this time the robot picked an object from the box and moved it either to the Near or to the Far position. A red fixation LED inserted in the middle of the object was then illuminated, which the monkey had to fixate (keeping the gaze within a ± 3.5 degrees—Monkey D—or ± 5 degrees—Monkey Y—throughout the trial). After 500 ms, a white LED illuminated the object from within for a variable amount of time (350–1500 ms). If the red fixation LED did not dim until the end of the trial (Fix trials), the monkey’s gaze remained inside the electronically defined window, and the hand remained in the resting position, the animal received a juice reward. In the other half of the trials, the red LED in the middle of the object dimmed (Go cue—Grasp trials), which was the signal for the monkey to lift his hand from the resting position, reach and pull the object for 300 ms (holding time) in order to obtain a reward. Note that when the object appeared at the Near position, the animal could not predict whether the trial would be a Fix trial or a Grasp trial up to the moment of the dimming of the red fixation LED.

All conditions (Fix Near, Fix Far and Grasp) were randomly interleaved in blocks of 10 trials per object type.

All data analysis was performed in Matlab (Mathworks). For each trial, the baseline firing rate was calculated from the mean activity recorded in the 300 ms interval preceding Light onset (white LED). For the Fix trials, we then calculated the net neural responses by subtracting the baseline activity from the mean activity observed between 40 and 600 ms after Light onset, and averaged the activity across trials regardless of trial length (since the fixation period varied between 350 and 1500 ms). We tested visual responsivity by means of *t* tests (*p* < 0.05) comparing the baseline activity to the activity in the period after Light onset. All neurons included in the current study were active in the Fixation task, which was the topic of this study, and in the VGG task (described in^25^).

For the Fix conditions, both cell-by-cell and population analysis were performed to quantify distance and object selectivity. For every responsive neuron, we computed a two-way ANOVA with factors *[distance]* and *[object] and* counted the number of cells with a significant main effect of distance, a significant main effect of object, or a significant *[distance* × *object]* interaction. For all ANOVAs with a significant main effect or interaction effect, we calculated post-hoc tests using the *multcompare* function, with default Critical Value—Tukey–Kramer in Matlab.

We plotted the averaged population net response to each object (ranked from Best to Worst) at the Near and at the Far distance, for each area. Since we noticed differences in the neuronal selectivity over time after stimulus onset, we arbitrarily defined two visual epochs in which all the following statistical analyses were performed, i.e. Early—from 40 to 200 ms after Light onset—and Late—from 200 to 400 ms.

Next, we investigated the effect of viewing distance by first determining the preferred viewing distance (i.e., Best) for every neuron based on the average net responses at the two viewing distances.

To compare neuronal selectivity for objects and distance across areas, we calculate d′ values as:

$${\text{D}}^{\prime } = {\text{mean}}_{{{\text{pref}}}} {-}{\text{mean}}_{{{\text{nonpref}}}} /\surd \left( {{\text{variance}}_{{{\text{pref}}}} + {\text{variance}}_{{{\text{nonpref}}}} } \right)/2$$

where *pref* and *nonpref* represented preferred and nonpreferred object or distance, similar to Decramer et al. (2019), based on the net neural responses in the interval from 40 to 600 ms after stimulus onset. To compare the distance selectivity across areas we also calculated raw d′, which was defined as:

$${\text{D}}^{\prime } = {\text{mean}}_{{{\text{Near}}}} {-}{\text{mean}}_{{{\text{Far}}}} /\surd \left( {{\text{variance}}_{{{\text{Near}}}} + {\text{variance}}_{{{\text{Far}}}} } \right)/2$$

Likewise, we calculated d′ values on the responses to the best object at the Near position compared to the response to the same object at the Far position (same interval), as well as on the responses to the best object at the Far position versus the same object at the Near position.

Finally, to assess the neural selectivity for objects with identical retinal size, we compared the average net response to the best small object presented at the Near position to the average net response to the same shaped large object at the Far distance (test for significance in the two epochs—*t* test *p* < 0.05). The d′ values were calculated on the average net responses to the preferred small object Near (i.e., eliciting the highest response) versus the large object of the same shape Far (interval 40 ms to 600 ms after stimulus onset).

All methods were carried out in accordance with ARRIVE guidelines.

## Results

### Object and distance selectivity

**Figure 2:**
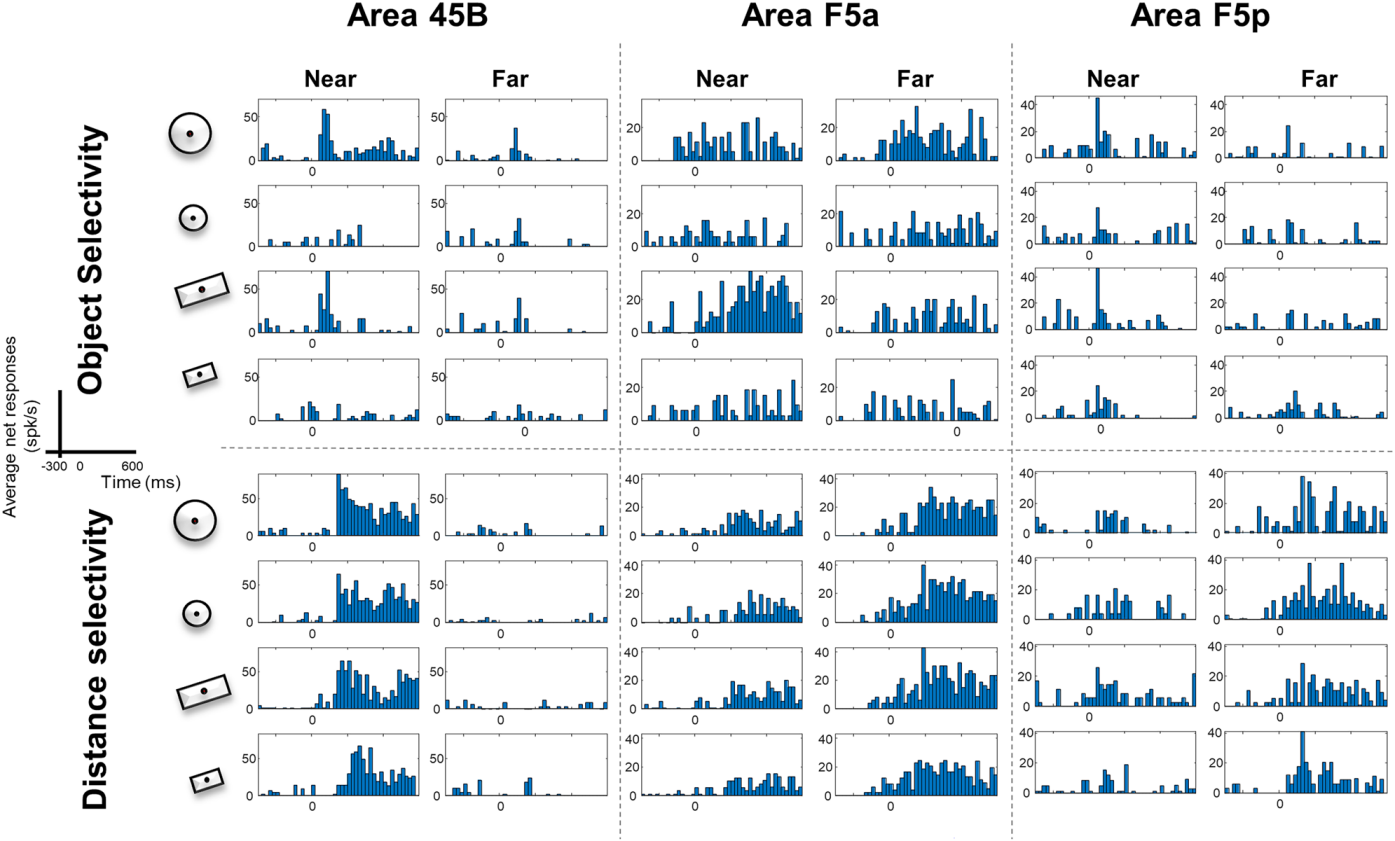
Example neurons in area 45B, F5a and F5p, from Monkey D and Y. In the upper panel, three neurons showing Object selectivity (from left to right, preference for large objects, large plate and large sphere, all from Monkey D except for F5a’s); in the lower panel, three example neurons of Distance selective neurons (from left to right, preference for Near, Far, Far, all from Monkey D). All example cells are separated by dashed lines. All neurons are aligned at light onset.

All data included in this analysis were recorded in Fix trials, which were randomly interleaved with Grasp (VGG) trials. Since the results were qualitatively similar between the two animals, we combined the data sets for all analyses, and provided the data separately for the two animals in Extended data. All neurons included (Monkey D: 45B, n = 57; F5a, n = 45; F5p, n = 44; Monkey Y: 45B, n = 57; F5a n = 44; F5p n = 32—total number of neurons: 279) responded significantly to at least one object during fixation after Light onset. In each of the areas, we observed both Object- and Distance-selective neurons. The example neurons in Fig. 2 illustrate the typical object (upper panel) and distance (lower panel) selectivity we observed in each of the areas. The example neuron of area 45B was clearly object-selective at the Near distance but not at the Far distance (two-way ANOVA, *object* × *distance* interaction, F = 4.22, *df* = 3, *p* = 0.007). In F5a, the object selectivity was generally weaker, and the responses evolved more slowly, as in the example neuron (middle column), whereas F5p neurons often showed transient responses to light onset with some object selectivity (right column). The lower panel of Fig. 2 illustrates examples of distance selectivity in the three areas without clear object selectivity. The example neuron of 45B preferred the Near position (two-way anova main effect of distance, *p* = 1.25 × 10^–24^, F = 168.73, *df* = 1, η = 2.79%), whereas the example neurons of F5a and F5p preferred the Far position.

The example neurons in Fig. 2 also illustrate the different response profiles in the three areas. While area 45B neurons had a fast increase of the firing rate after light onset, neurons in area F5a showed a slower ramping up of the response without any brisk increase after light onset. Neurons in area F5p, instead, had an intermediate profile between 45B and F5a. To quantify the effects of Object, Distance and the interaction between these two factors, we performed a two-way ANOVA on the responses of each neuron. We observed a significantly higher proportion of object-selective neurons in area 45B compared to F5a and F5p (main effect of Object significant at *p* < 0.05 in 41% of the neurons in 45B, compared to 20% in F5a, *p* = 7.4 × 10^–4^; and 24% in F5p, chi^2^ = 12.4, *p* = 2.0 × 10^–3^). The proportion of neurons with a significant effect of Distance did not differ among the areas (45B: 44%; F5a: 36% and F5p: 37%, Supplementary Table 1. chi^2^ = 1.60, *p* = 0.45).

**Figure 3:**
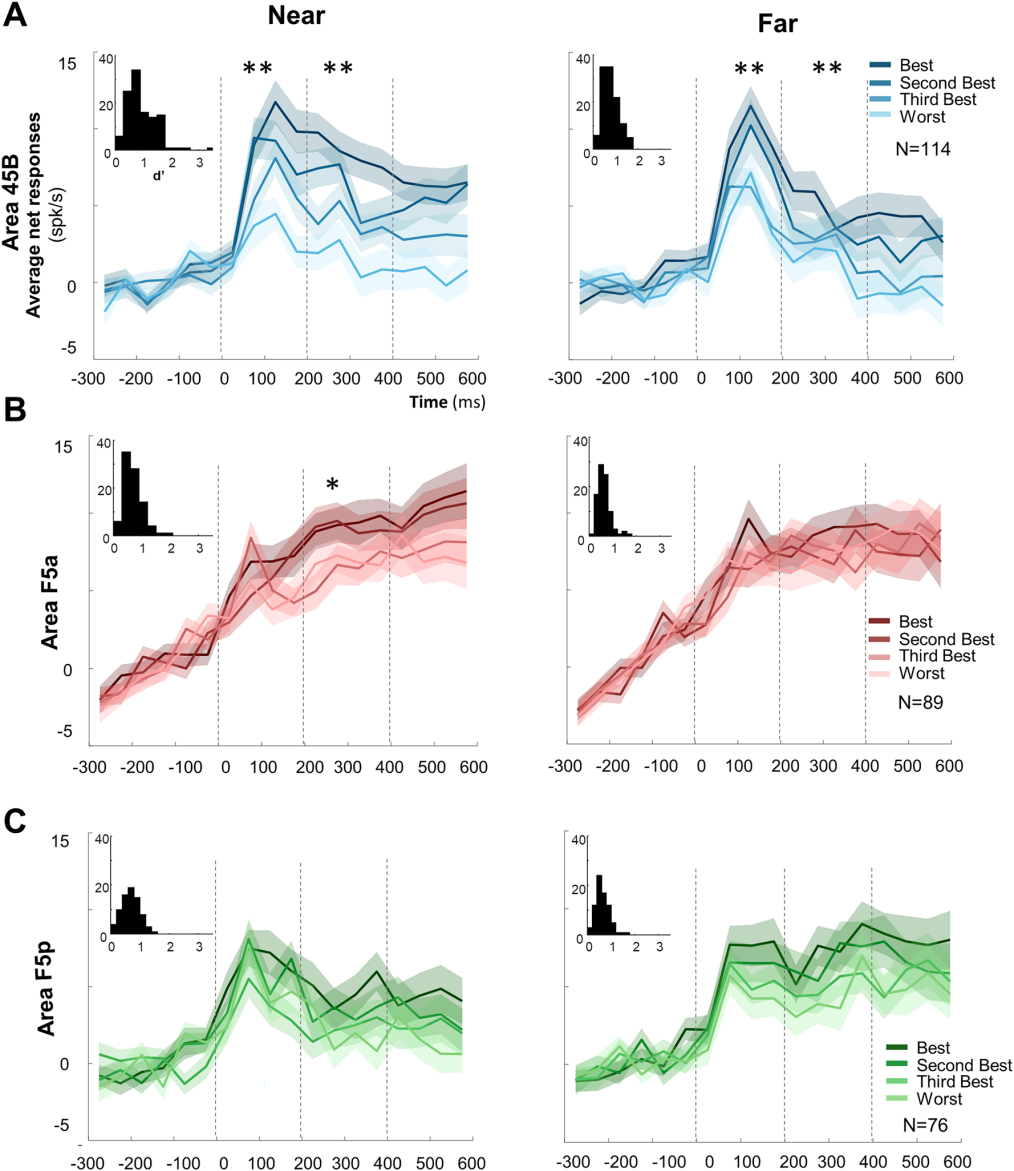
Object selectivity. Average ranked (on odd trials) population response of the even trials of object responsive neurons at Near and Far distance (one-way ANOVA). Average net response across monkeys in area 45B (blue shades; N = 114) (**A**), F5a (red shades; N = 89) (**B**) and F5p (green shades; N = 76) (**C**). Independently from the area, the darkest colors represent the Best object; progressively lighter colors represent lower ranking. Shadows of same color represent sem. The bin size was 50 ms. One asterisk indicates *p* < 0.05; two asterisks indicate *p* < 0.01. Insets indicate d′ distributions for object selectivity, at Near and Far distance, for each area.

To illustrate the object selectivity in each area, we ranked the objects for each responsive neuron based on the responses in the odd trials, and plotted the average response to the four objects in the even trials based on this ranking (Fig. 3). In area 45B, we observed significant differences between preferred and non-preferred objects in both epochs (Early 0–200 ms, and Late 200–400 ms) at both distances (one-way ANOVA, main effect of object *p* = 4.02 × 10^–5^, F = 7.86, *df* = 3, η = 4.96% and *p* = 2.50 × 10^–3^ F = 4.85, *df* = 3, η = 3.12% Near and Far for the Early epoch, respectively. *p* = 1.46 × 10^–6^, F = 10.28 *df* = 3, η = 6.39%, and *p* = 3.84 × 10^–3^ F = 2.82, *df* = 3, η = 1.84%, respectively Near and Far for Late epoch—see Supplementary Table 2 for detailed statistics). Conversely, we did not observe comparable significant differences across objects in the other two areas (at the Near distance F5a was only significant during the late epoch, *p* = 2.60 × 10^–2^ F = 3.13, *df* = 3, η = 2.59%, while F5p was never significant for both Near and Far distance, i.e., *p* > 0.05 at all-time epochs). A two-way ANOVA with factors [*object]* and *[area]* revealed that the object selectivity differed significantly between the three areas (main effect of *area*: *p* = 1.41 × 10^–5^, F = 11.40, *df* = 2, η = 3.87% for Near, and *p* = 1.10 × 10^–5^, F = 11.66, *df* = 2, η = 4.03% for Far). The object selectivity was stronger in 45B and F5p than in F5a at the Near distance (post-hoc test (*multcompare* function, with default Critical Value—Tukey–Kramer): 45B vs. F5a, *p* = 2.91 × 10^–4^; 45B vs. F5p, *p* = 0.65; F5a vs. F5p, *p* = 3.88 × 10^–5^) and stronger in 45B than in the other two areas at the Far distance (post-hoc tests: 45B vs. F5a, *p* = 1.13 × 10^–5^; 45B vs. F5p, *p* = 3.90 × 10^–3^; F5a vs. F5p, *p* = 0.48).

This difference in object selectivity between the three frontal areas was confirmed by an analysis of the d′ values (insets in Fig. 3). At the Near position, the average d′ (calculated on the responses to the preferred and nonpreferred object irrespective of size) was significantly higher in 45B compared to F5a and F5p (average d′ was 0.940, 0.689, 0.687, respectively; one way ANOVA, *p* = 9.70 × 10^–6^; post-hoc tests: 45B vs. F5a, *p* = 7.76 × 10^–5^; 45B vs. F5p, *p* = 1.4 × 10^–4^; F5a vs. F5p, *p* = 0.99). Similarly, at the Far position the average d′ was significantly higher in 45B than F5a and F5p (0.78, 0.62, and 0.63, respectively; one way ANOVA, *p* = 5.52 × 10^–4^; post-hoc test: 45B vs. F5a, *p* = 1.50 × 10^–3^; 45B vs. F5p, *p* = 5.10 × 10^–3^, F5a vs. F5p, *p* = 0.98).

To rule out the possibility that the object selectivity was induced by differences in eye movements between the four objects, we plotted the average horizontal position of the right eye for the four objects and the two viewing distances in Supplementary Fig. 1B. In both monkeys, the eye position signal deviated slightly after Light onset (and more pronounced in monkey Y and at the Near viewing distance), which could be partially caused by the pupil’s response to light onset. However, the eye traces were very similar across the four objects in our experiment (all differences were less than 0.6 deg in monkey D and less than 1 deg in monkey Y.

**Figure 4:**
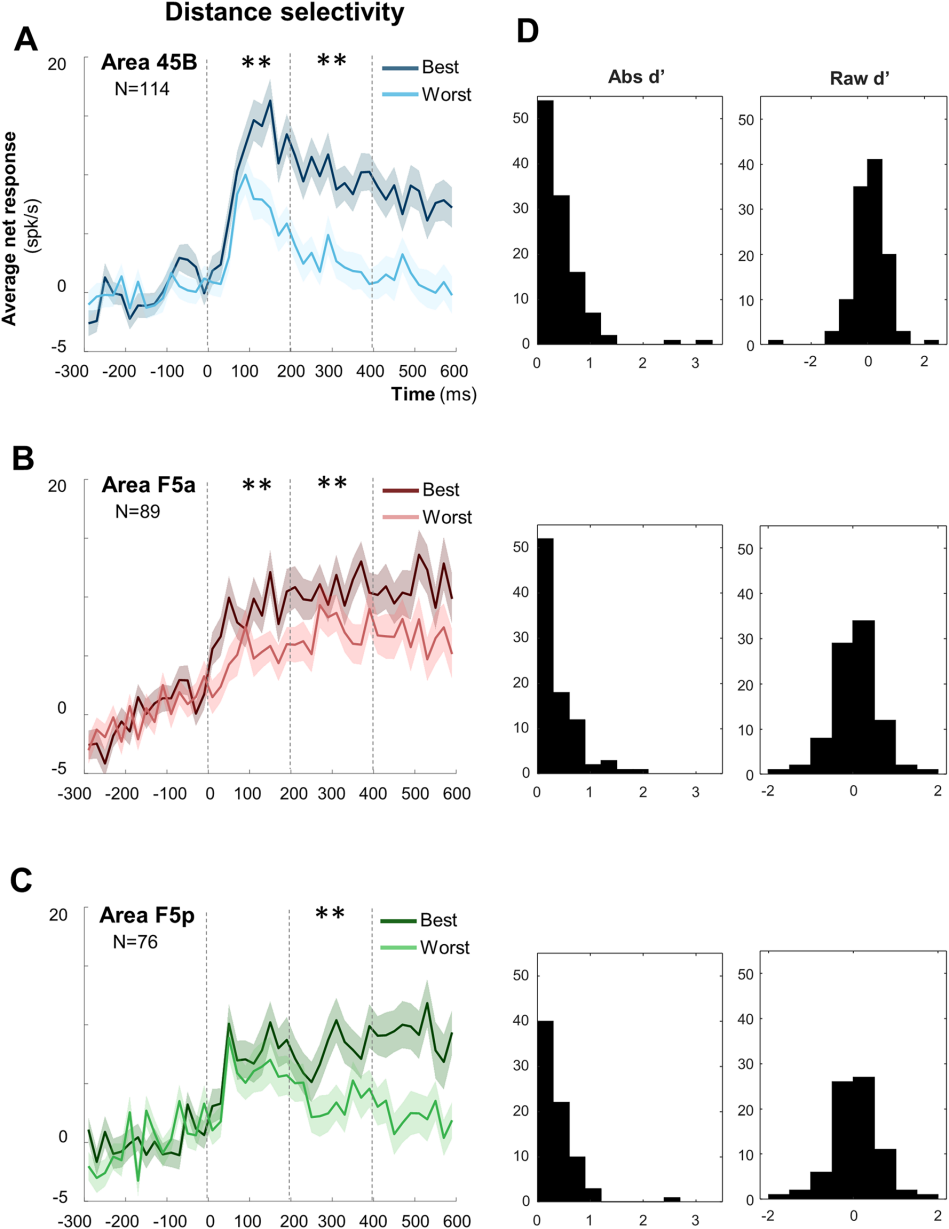
Distance selectivity comparing object responses at the Best and at the Worst distances. (**A**)–(**C**) Average distance selectivity of the even trials, previously selected on odd trials, comparing the same object at the two distances (bin size = 20 ms): darker colors represent the Best distance for the three areas (blue for 45B, red for F5a, and green for F5p); the lighter color shade indicates Worst distance. Shadows of same color represent sem. Double asterisks indicate statistical significance (*p* < 0.01). See also Supplementary Fig. 2, for Monkey D and Y, separately. (**D**) absolute and raw Distance selectivity d′ distribution for area 45B, F5a and F5p respectively.

Next, we investigated the effect of viewing distance by first determining the preferred viewing distance for every neuron based on the average net responses (interval 40—600 ms after stimulus onset) at the two viewing distances. Contrary to our expectations, we observed significantly more neurons preferring the near distance in area 45B (58%) and in area F5a (55%) than in F5p (39%, chi^2^ = 6.69, *p* = 0.035; Supplementary Table 3). To quantify the strength of the distance selectivity, we calculated d′_distance_ on the responses to all objects Near compared to all objects Far (Fig. 4D). All three areas showed robust distance selectivity, but only F5p showed an unexpected significant preference for the Far position (average d′ = -0.21 for F5p, *t* test: *p* = 4.69 × 10^–4^, compared to + 0.08 for 45B and + 0.04 for F5a; one-way ANOVA across areas, *p* = 1.7 × 10^–3^; post-hoc tests: 45B vs. F5a, *p* = 0.90; 45B vs. F5p, *p* = 1.60 × 10^–3^; F5a vs. F5p, *p* = 1.14 × 10^–2^). Overall, the strength of the distance selectivity quantified in the absolute d′ values was higher in 45B (one-way ANOVA, *p* = 9.87 × 10^–4^; mean absolute d′ = 0.43) compared to F5a (0.37, post-hoc test: 45B vs. F5a, *p* = 2.23 × 10^–2^) and F5p (0.36, post-hoc test 45B vs. F5p, *p* = 8.11 × 10^–4^; F5a vs. F5p, *p* = 0.58). In the average population responses (Fig. 4C), the distance selectivity in F5p was only significant in the late epoch of the response, whereas 45B and F5a were selective in both early and late epoch (Fig. 4A,B). (See Supplementary Table 4). Averaged across neurons and across the entire stimulus presentation interval, frontal neurons responded 79% (45B), 55% (F5a) and 74% (F5p) less to the preferred object at the worst distance compared to the same object at the best viewing distance.

**Figure 5:**
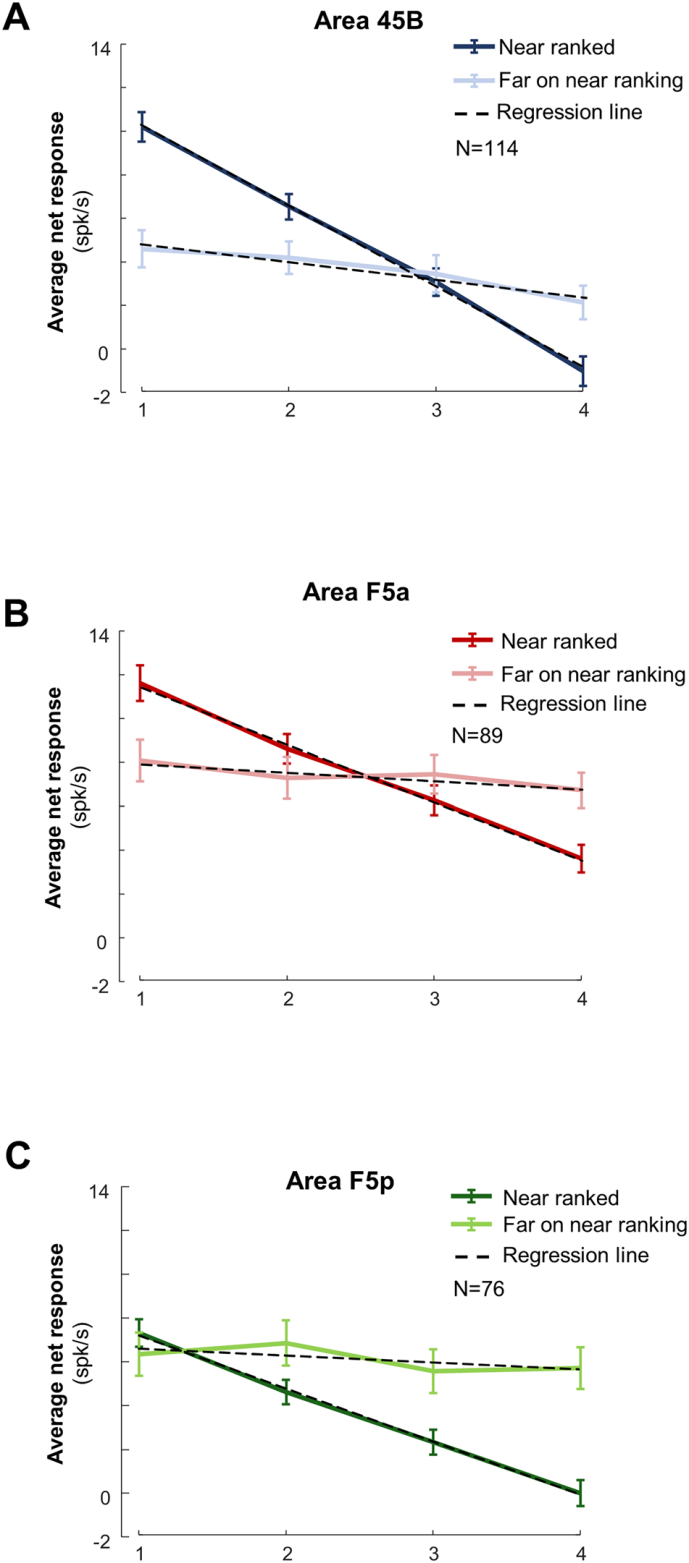
Ranking analysis: position tolerance across distances in depth. Average ranked responses at the Near position (dark color shades) and average responses to the same objects at the Far position (lighter color shades), for area 45B (blue; **A**), F5a (red; **B**) and F5p (green; **C**). Error bars of same colors represent sem. See also Supplementary Fig. 3, for Monkey D and Y, separately.

Viewing distance may not only affect the responses to the preferred object, but also the object preference of the neuron. In other words, is the object selectivity invariant across viewing distance? We first ranked the objects based on the average response of each neuron at the Near position, and then calculated the response to the same objects at the Far position. In all three areas, the slopes of the linear fits of the ranked responses at the Near position differed significantly from those of the responses at the Far position (Matlab routine fitlm—anova, Fig. 5 and Supplementary Table 5). Importantly, only 45B preserved some selectivity at the Far distance since the slope of the linear fit differed significantly from zero (slope − 0.81, CI [− 1.44 to − 0.19]), while the other two areas did not show any significant transfer of the object preference at the Far distance (Supplementary Table 5). Thus, only 45B neurons demonstrate some invariance of the object selectivity across viewing distance.

**Figure 6:**
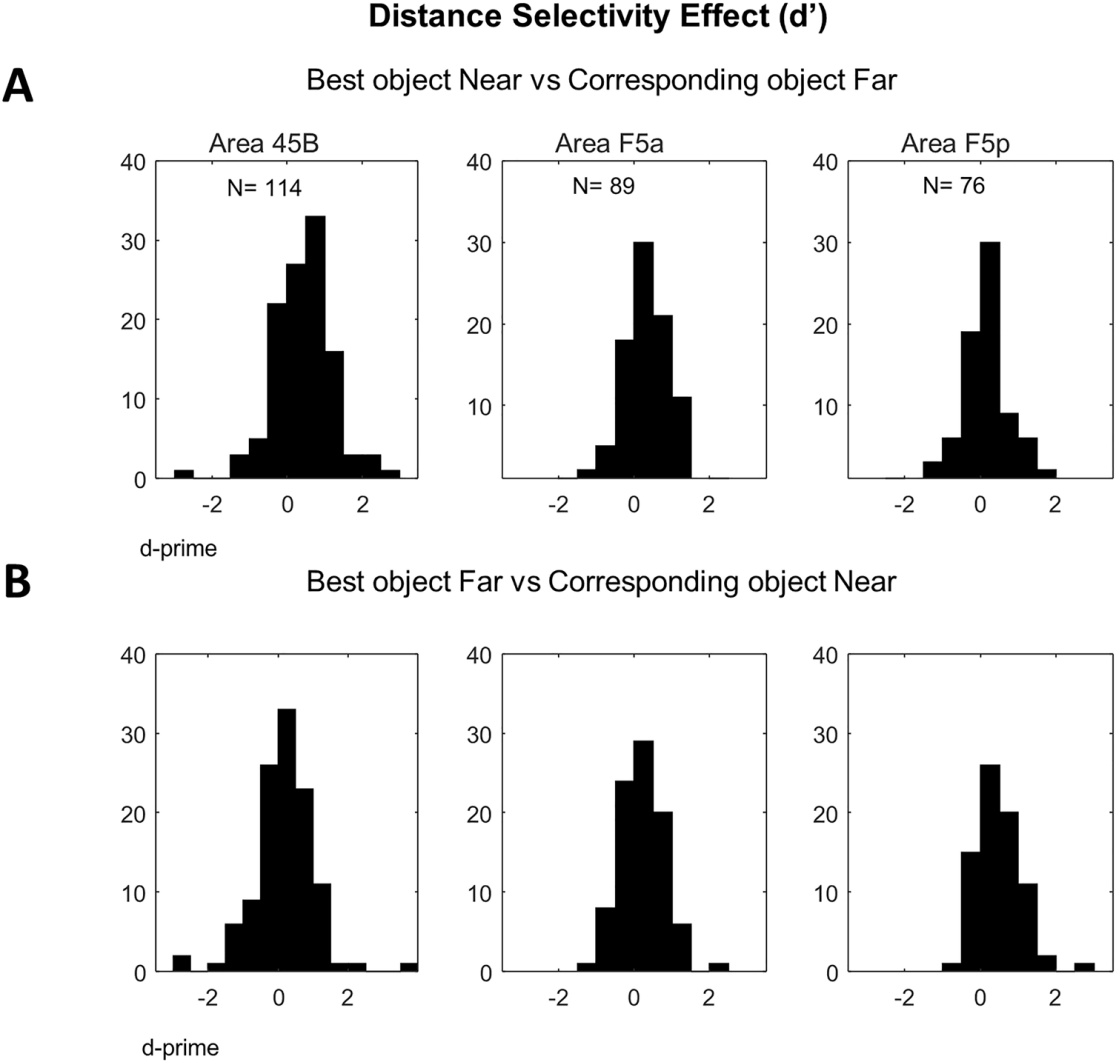
Distance selectivity effect (d′). (**A**) raw d′ for Best object Near versus Corresponding object Far for Area 45B, F5a and F5p, respectively; (**B**) raw d′ Best object Far versus Corresponding object Near for Area 45B, F5a and F5p, respectively.

To quantify the distance effect on the responses to the preferred object alone, we calculated d′ values for each neuron on the responses to the preferred object Near versus the same object Far (d′_Near_), and separately d′ values on the responses to the preferred object Far versus the same object Near (d′_Far_, Fig. 6). The three frontal areas differed significantly when selecting the preferred object at the Near position (one-way ANOVA on d′_Near_, *p* = 5.20 × 10^–3^; post-hoc tests: 45B vs. F5a, *p* = 0.31; 45B vs. F5p, *p* = 3.60 × 10^–3^; F5a vs. F5p, *p* = 0.18), with 45B showing stronger distance selectivity (average d′_Near_ = 0.46) than F5p (0.12, Fig. 6A). This difference was also significant when selecting the preferred object at the Far position (one-way ANOVA on d′_Far_, *p* = 4.0 × 10^–3^; post-hoc tests: 45B vs. F5a, *p* = 0.70; 45B vs. F5p, *p* = 2.80 × 10^–3^; F5a vs. F5p, *p* = 0.04; Fig. 6B).

**Figure 7:**
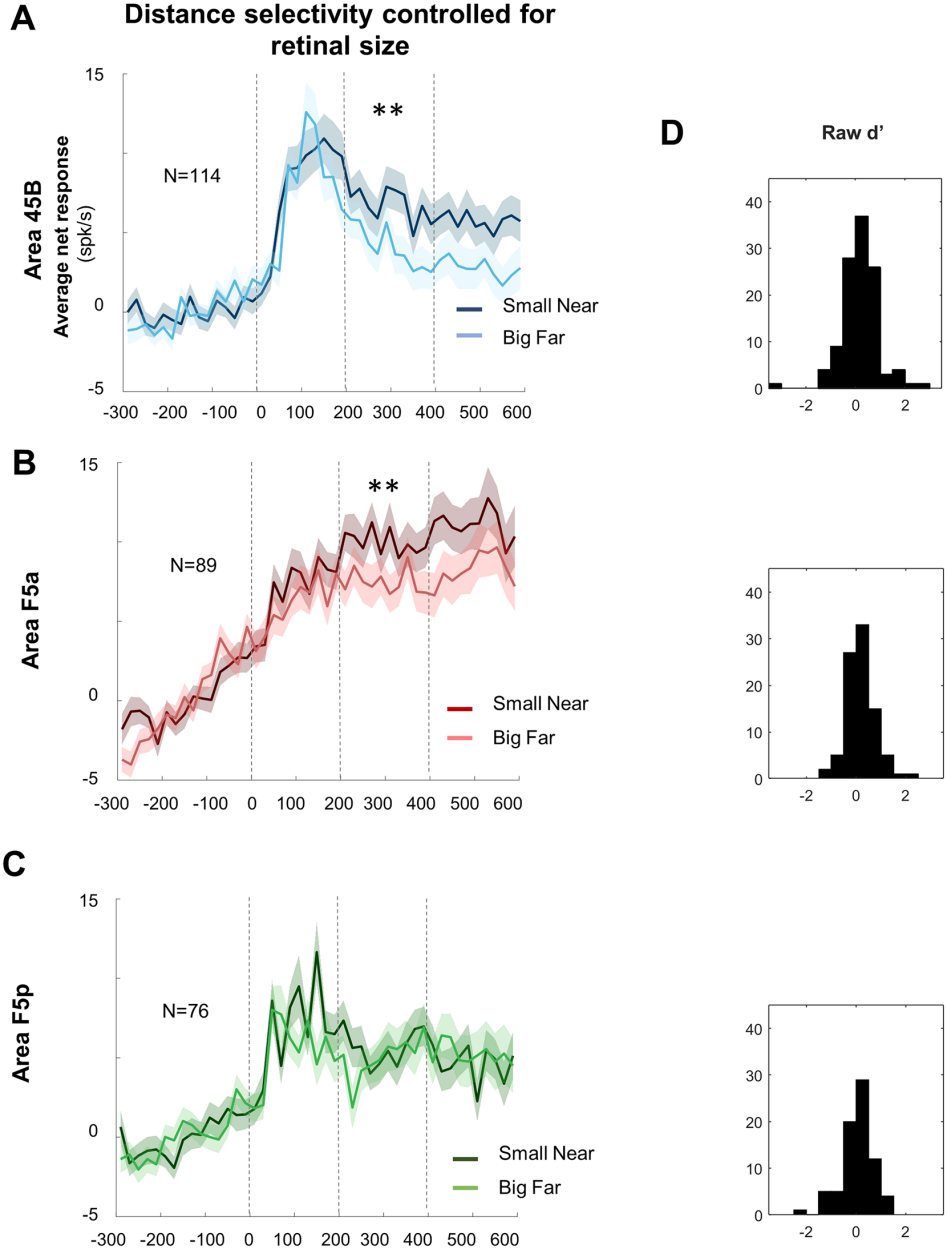
Distance selectivity controlled for retinal size. Average population response of objects with same retinal size for area 45B (**A**), F5a (**B**), and F5p (**C**—i.e. Small Near object = Large Far object). Dark colors represent small objects presented at Near position, while lighter colors represent large objects presented at Far conditions (blue, red, and green respectively). Shadows of same color represent sem. Bin size = 20 ms. Double asterisks indicate statistical significance (*p* < 0.01). See also Supplementary Fig. 4 for Monkey D and Y, separately. In (**D**) raw Retinal size selectivity d′ distribution for area 45B, F5a and F5p respectively.

Presenting the same object at the two positions also introduces a change in retinal size (Fig. 1C), which may influence the neuronal responses. To investigate the effect of viewing distance for objects with identical retinal size, we compared the average net responses to the small object at the Near distance with those to the same shaped large object at the Far distance (Fig. 7 and Supplementary Fig. 4). Only area 45B and F5a preserved a significant preference for the Near position during the Late epoch (*t* test, *df* = 113, *p* = 6.40 × 10^–3^ and *df* = 88, *p* = 1.80 × 10^–3^, respectively), but not in the Early epoch (*df* = 113, *p* = 0.23 for 45B and *df* = 88, *p* = 0.07 for F5a). In contrast, F5p neurons did not distinguish between these two conditions in none of the epochs (*t* test, *df* = 75, *p* = 0.07 and *p* = 0.45 for Early and Late epoch, respectively). The difference among the three frontal areas was not present in the distributions of the d′ values calculated on the two objects with identical retinal size (i.e., small object Near compared to large object Far; one-way ANOVA on d′ _retinal size_, p > 0.05). However, the average d′ _retinal size_ was significantly positive for areas 45B (0.19, *t* test, *df* = 113, *p* = 7.20 × 10^–3^) and F5a (0.20, *t* test, *df* = 88, *p* = 1.80 × 10^–3^) but not for F5p (0.05, *t* test, *df* = 75, *p* = 0.46) in F5p (Fig. 7D).

## Discussion

Neurons in area 45B, F5a and F5p responded to the presentation of the object and were selective for viewing distance, but only 45B neurons were selective for both object and viewing distance. Contrary to our expectations, we observed a strong preference for the Near viewing distance in 45B and F5a and a preference for the Far viewing distance in F5p (when retinal size was not controlled). Even for objects with identical retinal size at the two viewing distances, 45B and F5a neurons preferred the Near viewing distance. These results suggest that both area 45B and F5a may play a role in visually guided object grasping.

The source of the distance selectivity we observed cannot be easily determined from our experiments. Vertical disparities can be a source of visual information about distance, but only for relatively large stimuli (covering more than 10 deg,^26^). Since our largest objects were only 12 deg in diameter, vertical disparities are an unlikely source for distance in our study. Vergence angle, in contrast, clearly covaried with object distance and could therefore be used to inform the frontal areas about the object’s position in space. However, we cannot exclude the possibility that our animals also used other cues such as the position of the robot arm to estimate object distance.

The neural coding of viewing distance has been studied in early visual areas^27^, in dorsal^28–30^ and in ventral stream areas in nonhuman primates^31^. However, very few studies have investigated the neural coding of distance in the context of a reaching or grasping task^32^ investigated the effect of binocular eye position in rostral parietal area PE on the reaching-related activity of individual neurons, and reported that a small subpopulation of neurons was influenced by viewing distance^15,16^ described joint selectivity for fixation distance and reach direction in the caudal parietal area V6A. In these studies, the fixation distance varied within the peripersonal space of the animal (i.e. less than 25 cm from the animal), and therefore no data were obtained for targets that appeared beyond reaching distance.

Area 45B neurons showed fast and selective visual responses to the object after Light onset. Previous studies described fMRI activations in this area evoked by 2D images of objects^33,34^, and selectivity of individual neurons for shapes and very small line fragments^13^, and zero order disparity^9^. We confirmed the involvement of these neurons in shape and object processing during grasping. Most 45B neurons were also significantly affected by viewing distance and showed a preference for Near even when correcting for retinal size. Our hypothesis was that neurons that encode objects in purely visual terms should not be affected much by the distance manipulation (for identical retinal size), while neurons in which the object response primarily represents the motor plan would respond less to objects that cannot be grasped (i.e., in the far trials, again keeping the visual input identical by presenting a larger object at the far distance). However, we observed the opposite effect: neurons in the more ‘visual’ area 45B preferred the near viewing distance whereas the more ‘motor’ area F5p showed a slight preference for the far presentations. This observation—together with their strong responses during visually-guided object grasping^25^—is compatible with the idea that area 45B neurons are important for processing object information during grasping.

Because of the visual properties of this area and the direct anatomical connection with pAIP^8^, we previously hypothesized that area 45B could be involved in oculomotor control, similar to the neighboring region FEF^13,35^, to guide eye movements towards specific parts of the object contour. However, we observed sustained object responses in the fixation epoch of a visually-guided grasping task, which do not seem to be consistent with pure oculomotor control since no saccade was required after obtaining fixation. Our results rather suggest that area 45B may have a role in object processing and eye-hand coordination when grasping objects under visual guidance. The strong distance selectivity with a preference for Near was also inconsistent with our initial hypothesis, and could not be explained as a vergence effect, as the eye position was stable after light onset. At least for the subpopulation of neurons preferring the peripersonal space, we cannot exclude the possibility that the distance effect we observed was related to the significance that the stimulus acquired when it was reachable, and therefore graspable. Our results are in accordance with^21^, who reported activations in prefrontal cortex in response to object presentation at reachable distances.

The F5a results were largely consistent with the known anatomical connectivity and neuronal properties of this area. Although we measured significant object selectivity in individual F5a neurons, our population of F5a neurons was only weakly object-selective, most likely because we only used a limited number of objects. Nevertheless, the F5a neurons we recorded were strongly affected by viewing distance, preferring the Near viewing distance even for stimuli with identical retinal size. Overall, these properties are consistent with the proposed position in the cortical hierarchy as ‘pre-premotor cortex’^35^, receiving visual inputs from AIP and transmitting information to F5p. Note that—in contrast to F5p neurons—45B and F5a neurons with different distance preferences behaved similarly in the VGG task, which may be related to the primarily visual character of the responses in these two areas.

Previous studies have described the connectivity pattern of F5p as strongly motor-oriented, receiving input from F5a and projecting directly to primary motor^36,37^. Being part of the same pathway (pAIP → aAIP → F5a → F5p), several authors comparing object representations in AIP and F5p^11,12^ concluded that AIP provides a visual object description, while area F5p represents the same object in motor terms, i.e., the grip type necessary to grasp the object. As shown in^25^, Near preferring F5p neurons showed stronger peri-lift modulation than Far preferring neurons, suggesting a more prominent role in motor planning and execution of these neurons^38^ reversibly inactivated F5 (probably F5p) with muscimol, which induced a deficit in the preshaping of the hand during grasping. Because objects may be encoded in motor terms in F5p, we hypothesized that F5p would strongly prefer the Near distance but unexpectedly, a high number of neurons preferred the Far viewing distance. Our results seem to be in contrast to^21^, reporting Near preference in F4/F5p area. However, the ‘Far space’ distance for object presentation in^21^ was significantly larger than in our case (150 cm and 56 cm, respectively). Therefore, it is possible that our Far viewing distance was not long enough to reduce the visuomotor neural responses in F5p. Another difference with our study is that^21^ analyzed fMRI responses in monkeys, which may also include subthreshold modulations and presynaptic activity^39^. The unexpected preference for the Far viewing distance in F5p may be related to its role in reaching movements^40^, in line with the connectivity of F5p with dorsal premotor cortex and parietal area PG^37^.

Our results are important for the interpretation of the organization of the parieto-frontal grasping network. In pAIP, visual information is transmitted along two parallel pathways: towards aAIP, and then to F5a and F5p, and directly to 45B^8^. The fact that 45B neurons preferred objects in peripersonal space even after equating retinal size, together with its direct input from posterior AIP, suggests that this area may also play a role in object processing during visually-guided object grasping. Recently, we showed that area 45B is causally related to object grasping using reversible inactivations^25^, suggesting that eye-hand coordination (monitoring the position of the own hand approaching the object) may be supported by 45B neurons.

Our data suggest a much more complex role of 45B in the network rather than directing saccades towards object contours. The strong visual responses and the surprising preference for the Far viewing distance of area F5p suggest that the visuomotor object representation in F5p was still activated when we presented objects beyond reaching distance. The presence of a transparent barrier interposed between the monkey and the object could have decreased or silenced the F5p response to objects located both at the Near and Far distances in a similar way as in^17^.

## Supplementary Information

Supplementary Information.
