# Supplementary material for: Effect of viewing distance on object responses in macaque areas 45B, F5a and F5p
Source: Sci Rep. 2022 Oct 3;12:16527. doi: 10.1038/s41598-022-18482-4 (PMC9530235; doi:10.1038/s41598-022-18482-4)
Supplement: Supplementary file 1 — Supplementary Information. [file 41598_2022_18482_MOESM1_ESM.pdf]

Table 1. Object, Distance and Interaction effects

	Area 45B	Area F5a	Area F5p
**Object**	47 (41%)	18 (20%)	18 (24%)
**Distance**	50 (44%)	32 (36%)	28 (37%)
**Interaction**	23 (19%)	12 (13%)	9 (12%)

Table 1. Neuron count for Object, Distance and Interaction effects. Numbers of neurons with a significant effect of Object, Distance and Interaction in 45B (N = 114), F5a (N = 89) and F5p (N = 76).

Table 2. Object Selectivity

	Near								Far							
	**P-val**		**F**		**df**		**η (%)**		**P-val**		**F**		**df**		**η (%)**	
	Early	*Late*	*Early*	*Late*	*Early*	*Late*	*Early*	*Late*	*Early*	*Late*	*Early*	*Late*	*Early*	*Late*	*Early*	*Late*
**Area 45B**	4.02×10^{-5}	1.46×10^{-6}	7.86	10.28	3	3	4.96	6.39	2.50×10^{-3}	3.84×10^{-3}	4.85	2.82	3	3	3.12	1.84
**Area F5a**	0.24	2.60×10^{-2}	1.41	3.13	3	3	1.19	2.59	0.5	0.81	0.79	0.32	3	3	0.67	0.27
**Area F5p**	0.09	0.12	2.18	1.94	3	3	2.13	1.9	0.13	0.16	1.92	1.72	3	3	1.89	1.69

Table 2. Statistics on Object selectivity. Object selectivity at Near and Far viewing distances for all responsive neurons in area 45B, F5a and F5p: detailed statistics (One-way Anova – P-val, F, df, η; See Figure 3).

Table 3. Distance preference

	45B		F5a		F5p	
	**Near**	**Far**	**Near**	**Far**	**Near**	**Far**
Large	42	23	24	18	14	24
Small	24	25	25	22	16	22
**Total**	66 (58%)	48 (42%)	49 (55%)	40 (45%)	30 (39%)	46 (61%)

Table 3. Neuron count on Distance selectivity, divided in Large and Small size. Numbers of neurons with a significant effect of Distance, grouped in large and small sized objects. Respectively, 45B (N = 114), F5a (N = 89) and F5p (N = 76).

Table 4. Distance selectivity statistics (t-test) divided by Epoch

	**Early**	**Late**
**Area 45B**	1.98×10^{-7} (df =113)	3.62×10^{-9} (df =113)
**Area F5a**	4.49×10^{-4} (df =88)	2.30×10^{-4} (df =88)
**Area F5p**	0.13 (df =75)	3.82×10^{-4} (df =75)

Table 4. Distance selectivity statistics (t-test) divided by epoch (Early and late).

Table 5. Slopes of the regression line

	45B	F5a	F5p
**Near ranked**	-3.71 (-4.09– -3.33)	-2.65 (-3.03 – -2.26)	-2.45 (-2.70 – -2.20)
**Far on Near ranking**	-0.81 (-1.44 – -0.19)	-0.40 (-0.96 – 0.18)	-0.35 (-1.37 – 0.68)
**Matlab routine on fitlim**			
**ANOVA - Interaction effect** [Object rank x Distance rank]	6.96×10^{-5} (df = 1; F = 290.20)	1.43×10^{-4} (df = 1; F = 201.49)	1.00×10^{-3} (df = 1; F = 73.70)

Table 5. Ranking analysis statistics. Slopes (spikes/sec/stimulus rank) and confidence intervals of the regression lines of the average Near ranked and on the Far on Near ranking responses, divided per area (45B, N =114; F5a, N = 89; F5p, N = 76; See Figure 5). Statistics (Matlab fitlim routine -anova, Interaction effect between [Object rank x Distance rank]): df, F and p-value.

Supplementary Figure 1

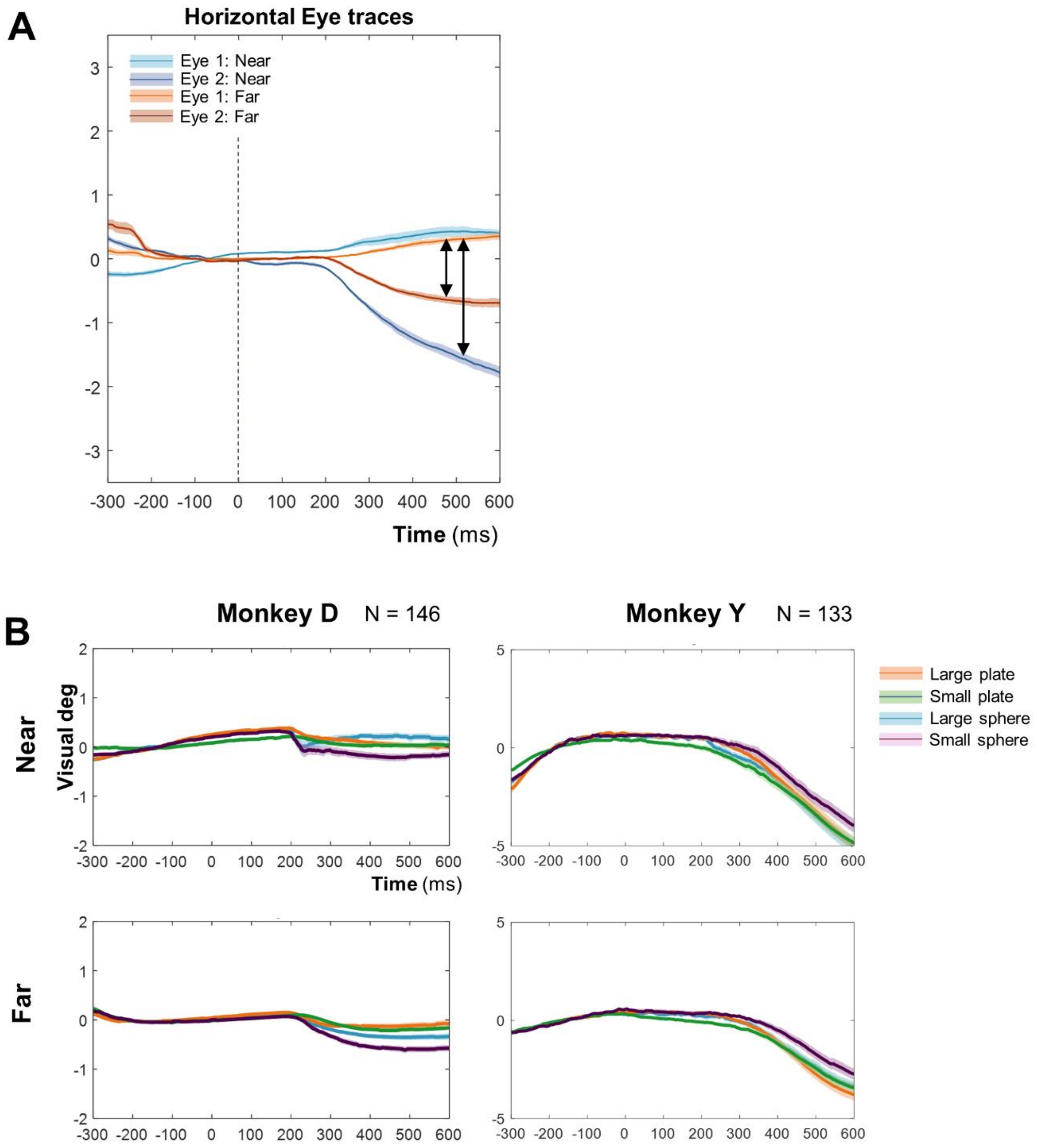

Figure 1. Eye traces. A. Binocular eye traces at Near and Far distances (Monkey Y). B. Average horizontal position of the right eye for the four objects used (Blue: Large sphere; Orange: Large plate; Green: Small plate; Purple: Small sphere), at the near and far viewing distance and separately for the two monkeys (Monkey D: left, Monkey Y: right panels).

Supplementary Figure 2

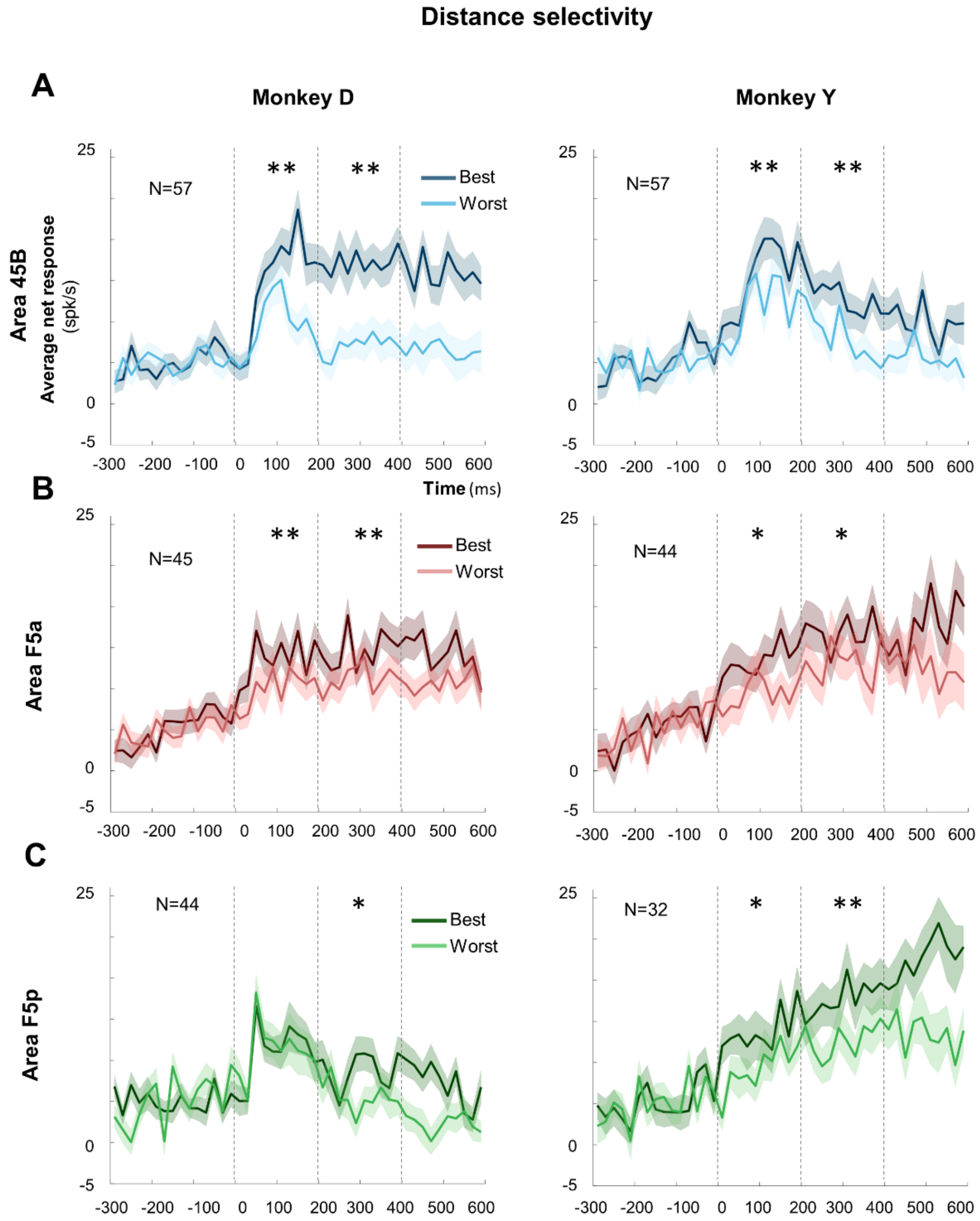

Figure 2. Distance selectivity comparing object responses at the Best and at the Worst distances, divided per monkey. Average distance selectivity of the even trials, previously selected on odd trials, comparing the same object at the two distances (bin size = 20 ms) for Monkey D (left) and Monkey Y (right). Darker colors represent the Best distance for the three areas: blue for 45B (A), red for F5a (B), and green for F5p (C); the lighter color shade indicates Worst distance. Shadows of same color represent sem. One asterisk indicates $p<0.05$; two asterisks indicate $p<0.01$.

Supplementary Figure 3

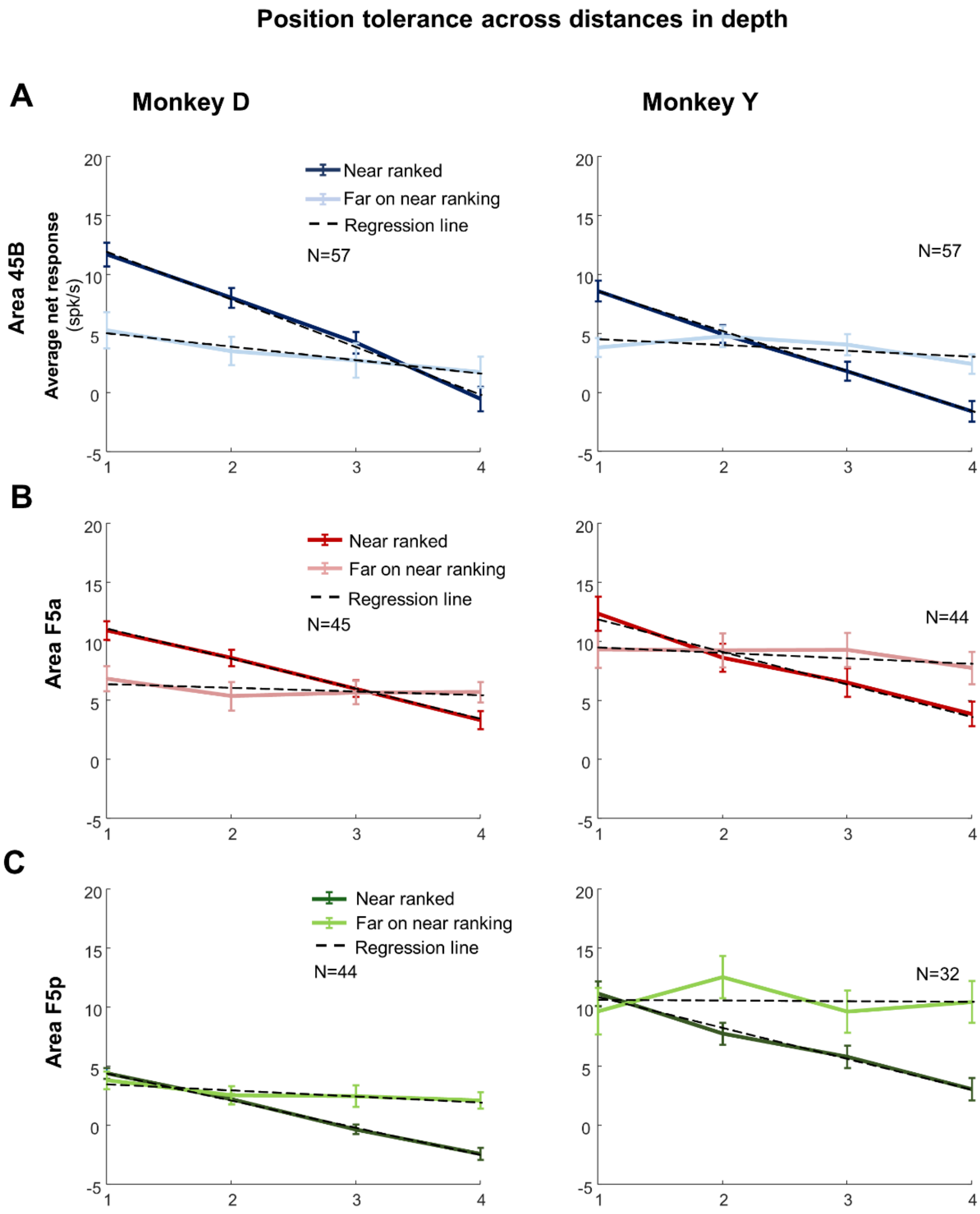

Figure 3. Ranking analysis: position tolerance across distances in depth, divided per monkey. Average ranked responses at the Near position (dark color shades) and average responses to the same objects at the Far position (lighter color shades) in Monkey D (left) and Monkey Y (right), for area 45B (blue; A), F5a (red; B) and F5p (green; C). Error bars of same colors represent the standard error of the mean.

Supplementary Figure 4

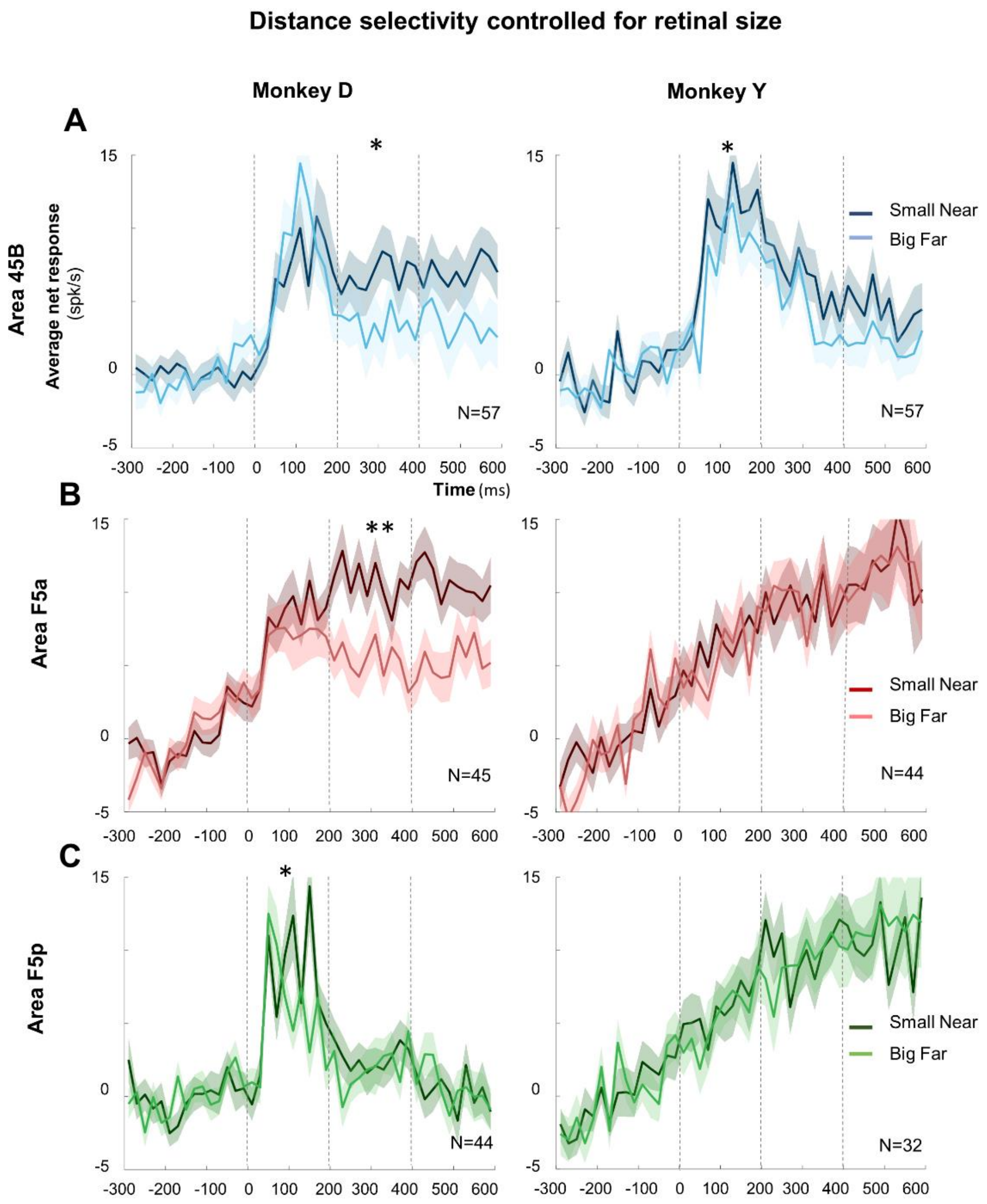

Figure 4. Distance selectivity controlled for retinal size, divided per monkey. Average population response of objects with same retinal size in Monkey D (left) and Monkey Y (right), for area 45B (A), F5a (B), and F5p (C - i.e., Small Near object = Large Far object). Dark colors represent small objects presented at Near position, while lighter colors represent large objects presented at Far conditions (blue, red, and green respectively). Shadows of same color represent sem. Bin size = 20 ms. One asterisk indicates $p<0.05$; two asterisks indicate $p<0.01$; Two asterisks correspond to $p<0.01$.
